# Trichostrongyloid nematodes in ruminants of northern Iran: prevalence and molecular analysis

**DOI:** 10.1186/s12917-021-03086-3

**Published:** 2021-12-04

**Authors:** Hedayat Hosseinnezhad, Meysam Sharifdini, Keyhan Ashrafi, Zahra Atrkar Roushan, Hamed Mirjalali, Behnaz Rahmati

**Affiliations:** 1 Department of Medical Parasitology and Mycology, School of Medicine, Guilan University of Medical Sciences, Rasht, Iran; 2 Department of Biostatistics, School of Medicine, Guilan University of Medical Sciences, Rasht, Iran; 3 Foodborne and Waterborne Diseases Research Center, Research Institute for Gastroenterology and Liver Diseases, Shahid Beheshti University of Medical Sciences, Tehran, Iran

**Keywords:** Trichostrongyloid nematodes, Ruminants, ITS2, Molecular analysis, Iran

## Abstract

**Background:**

This study was carried out to investigate the prevalence and analyze the molecular characteristics based on the internal transcribed spacer (ITS) 2 region of the ribosomal RNA (RNA) gene of trichostrongylid nematodes in different ruminants from Guilan province, northern of Iran.

**Methods:**

The gastrointestinal tracts of 144 ruminants including 72 cattle, 59 sheep, and 13 goats were collected from an abattoir in Guilan province during July to September 2018. After isolation the helminths, male specimens were identified based on morphological parameters. PCR and partial sequencing of the ITS2 fragment were conducted. After phylogenetic analysis, the intraspecific and interspecific differences were calculated.

**Results:**

The prevalence of total infections with the nematodes was 38.9, 74.6 and 84.6% among cattle, sheep and goats, respectively. Eleven species of trichostrongylid nematodes including *Haemonchus contortus*, *Marshallagia marshalli*, *Trichostrongylus axei*, *T. colubriformis*, *T. vitrinus*, *Ostertagia trifurcata*, *Teladorsagia circumcincta*, *Marshallagia occidentalis*, *O. lyrata*, *O. ostertagi*, and *Cooperia punctate* were recovered from the ruminants. The most prevalent trichostrongyloid nematodes in cattle, sheep and goats were *O. ostertagi* (26.4%), *M. marshalli* (64.4%) and *T. circumcincta* (69.2%), respectively. Phylogenetic tree was discriminative for Trichostrongylidae family, while phylogenetic analysis of the ITS2 gene represented low variations and no species identification of Haemonchidae and Cooperiidae families.

**Conclusions:**

This study suggests the high prevalence and species diversity of trichostrongyloid nematodes in different ruminants, indicating the importance of implement antiparasitic strategies in north regions of Iran. As well, this study showed that the ITS2 fragment is not a discriminative marker for Haemonchidae and Cooperiidae families, and investigation of other genetic markers such as mitochondrial genes would be more valuable for better understanding of their phylogenetic relationships.

## Background

Helminthic parasites cause long-term chronic infections associated with significant morbidity and mortality rates in both human and animal hosts. These infections lead to reduced productivity of ruminants in many countries [1]. There are several genera and an enormous number of species in super family Trichostrongyloidea, which are primarily parasites of the gastrointestinal tract of the animals with a worldwide distribution. Trichostrongyloid nematodes such as *Trichostrongylus* (Trichostrongylidae)*, Haemonchus* (Haemonchidae)*, Ostertagia* (Haemonchidae), *Nematodirus* (Molineidae), *Marshallagia* (Haemonchidae) and *Cooperia* (Cooperiidae), mostly live in abomasum and the first part of the small intestine in ruminants [2]. Actually, infection with these parasites leads to loss of meat, wool, and milk production and decreased feed intake, weight gains and growth rate [3]. These parasites also can occur serious or even fatal manifestations in ruminants and other grazers. Among the super family, *H. contortus* is one of the most pathogenic blood sucking nematodes, which causes severe anemia, hypoproteinemia, edema, and even death in ruminants [4]. Transmission of these nematodes is mostly through the ingestion of infective larvae in contaminated pasture and water [5]. The epidemiology of gastrointestinal helminths in domestic animals serves to improve prevention and control strategies, evaluation of risk factors, and decrease in production losses of the animals. In addition, these studies help to determine probable sources of animal and human infections [1, 6–8].

*Trichostrongylus* species are the potential zoonotic nematodes, which are prevalent in humans form some regions of the world, particularly in the Middle East, Far East, and some African countries [9–11]. Human trichostrongylosis has the highest infection rates and largest variety of species in Iran compared to other countries in the world due to close contact with ruminants in rural and nomadic populations [9]. Recent studies illustrated that Guilan province in north of Iran is one of most important endemic regions of human trichostrongylosis in Iran [7, 12–14]. Other genera of this super family including *H. contortus*, *M. marshalli*, *N. abnormalis*, *O. ostertagi* and *T. circumcincta* have been rarely reported in humans from different parts of world, especially in Iran [7, 9, 10, 15]. 

Recently, PCR based methods are used to identify species and phylogenetic analysis of different trichostrongyloid nematodes [16–19]. These studies have shown that the internal transcribed spacer 2 (ITS2) and the cytochrome c oxidase subunit 1 (*Cox1*) genes are useful targets for species differentiation and analyzing of the genetic variations [19–21]. The aim of this study was to determine the prevalence of the trichostrongyloid nematodes obtained from slaughtered indigenous ruminants in Guilan province, northern Iran, and to investigate of their genetic diversity using the ITS2 region of the rRNA gene.

## Methods

### Study area and samples collection

**Fig. 1:**
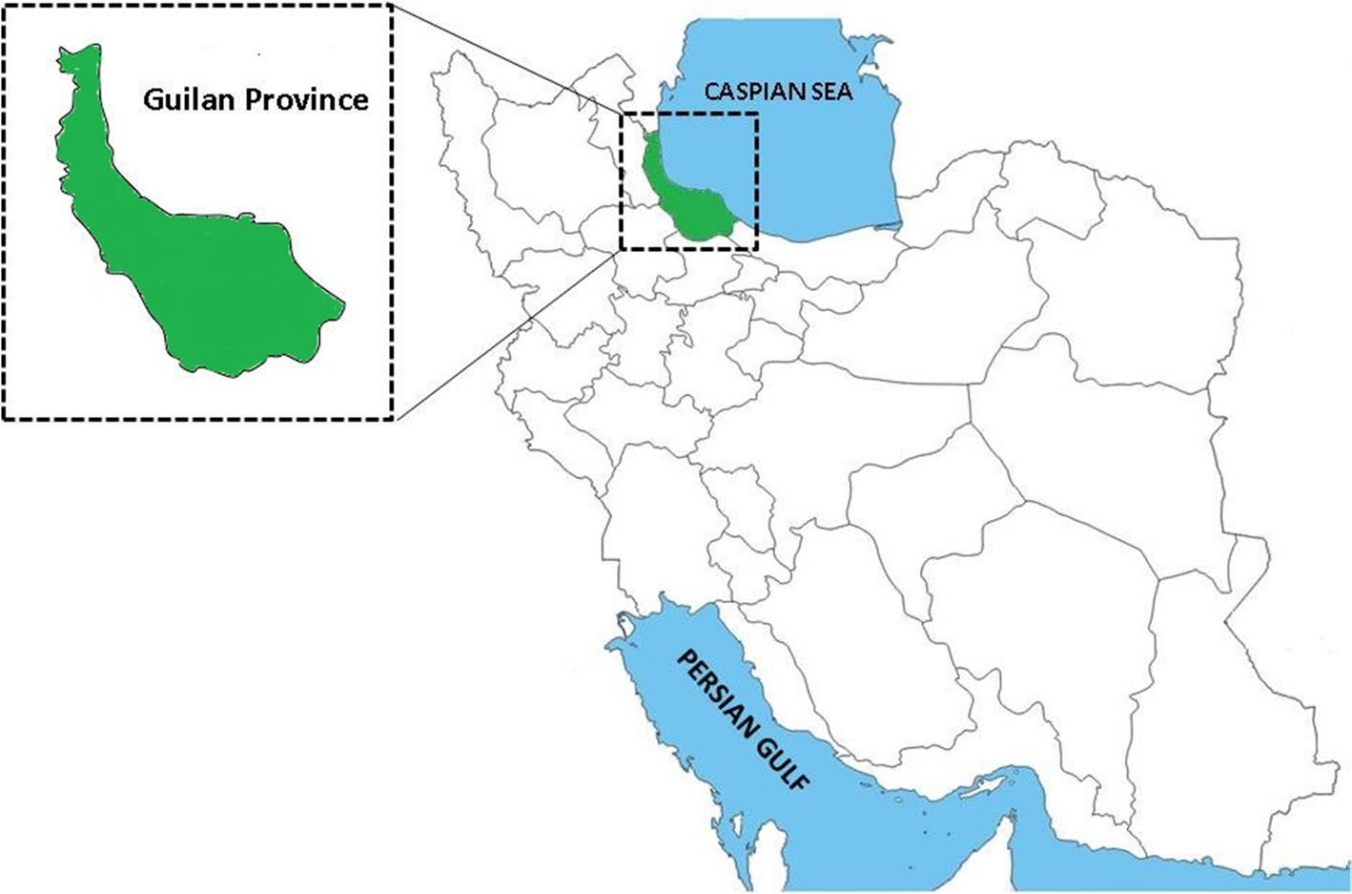
Map of Iran showing geographical location of Giulan Province

Guilan province is located in southwest coast of the Caspian Sea in the northern Iran (Fig. 1). It covers an area of 14,711 km^2^ (between 36° 36′ and 38° 27′ N, and between 48° 43′ to 50° 34′ E), and has temperature humid climate with mean annual rainfall of 1359 mm. The average seasonal temperature in Guilan province is 7.5 °C in winter, 18.5 °C in spring 24 °C in summer, and 13.5 °C in autumn. The average relative humidity is about 80%, which reaches to maximum in autumn and winter, and decreases in summer and spring [22]. In current study, a total of 144 ruminant gastrointestinal tracts from 72 cattle, 59 sheep, and 13 goats were collected from Talesh abattoir of Guilan province, northern of Iran, during July to September 2018. Animals all were local breed and samples were randomly collected from 10 to 15 animals in each sampling time (1 day in each week) for 12 weeks. The contents of abomasum and duodenum were washed using tap water runs through 20, 40, and 100 mesh sieves and examined for infectivity with trichostrongyloid nematodes by stereomicroscope. The obtained specimens were preserved in 70% ethanol for parasitological and molecular examinations. For morphological identification, the male nematodes were stained with Azo-carmine, and identified at the level of species using valid nematodes systematic keys [23, 24].

### DNA extraction and polymerase chain reaction (PCR) amplification

Total genomic DNA was extracted from one male worm of each species of trichostrongyloid nematodes in every kind of ruminants (*n* = 21) using a commercial DNA extraction kit (Yekta Tajhiz Azma, Tehran, Iran), according to the manufacturer’s instructions.

The ITS2 region was amplified using the primer pairs NC1 (5′- ACGTCTGGTTCAGGGTTGTT − 3′) and NC2 (5′- TTAGTTTCTTTTCCTCCGCT-3′) [25]. PCR conditions comprised an initial denaturing step of 95 °C for 6 min followed by 35 cycles of denaturation at 94 °C for 45 s, annealing at 60 °C for 90 s and extension at 72 °C for 60 s, and 72 °C for 5 min as a final extension. The amplification products were separated on 1.5% agarose gel electrophoresis, and visualized with UV transluminator (UVITEC, Cambridge, UK). Later, the PCR products were sent to a domestic sequencing company (Codon genetic company, Tehran, Iran) for sequence determination via the Sanger method.

### Phylogenetic analysis

The sequence results were edited and tr immed using Chromas version 2.01 (Technelysium Pty Ltd., Brisbane, Queensland, Australia), and compared to the GenBank submitted sequences using the BLAST programs and databases (http://www.ncbi.nlm.nih.gov/). The sequences of the ITS2 gene were submitted to the GenBank database (accession numbers: MN845160-MN845180). Phylogenetic analysis was performed with sequences obtained in the present study along with the reference sequences, which were deposited in the GenBank database, using Maximum-Likelihood algorithm and Tamura-3-parameter model in the MEGA 6.0 software. Bootstrap value was determined based on 1000 replications for evaluation of the phylogenetic tree reliability.

### Statistical analysis

Statistical analyses were performed using SPSS software version 18 (SPSS Inc., Chicago, Illinois, USA), and statistical tests including chi-squared (χ2) and Fisher’s exact tests were used to evaluate the association between trichostrongyloid nematode infections with different kinds of ruminants and sex of animals. A value less than 0.05 was considered statistically significant.

## Results

**Fig. 2:**
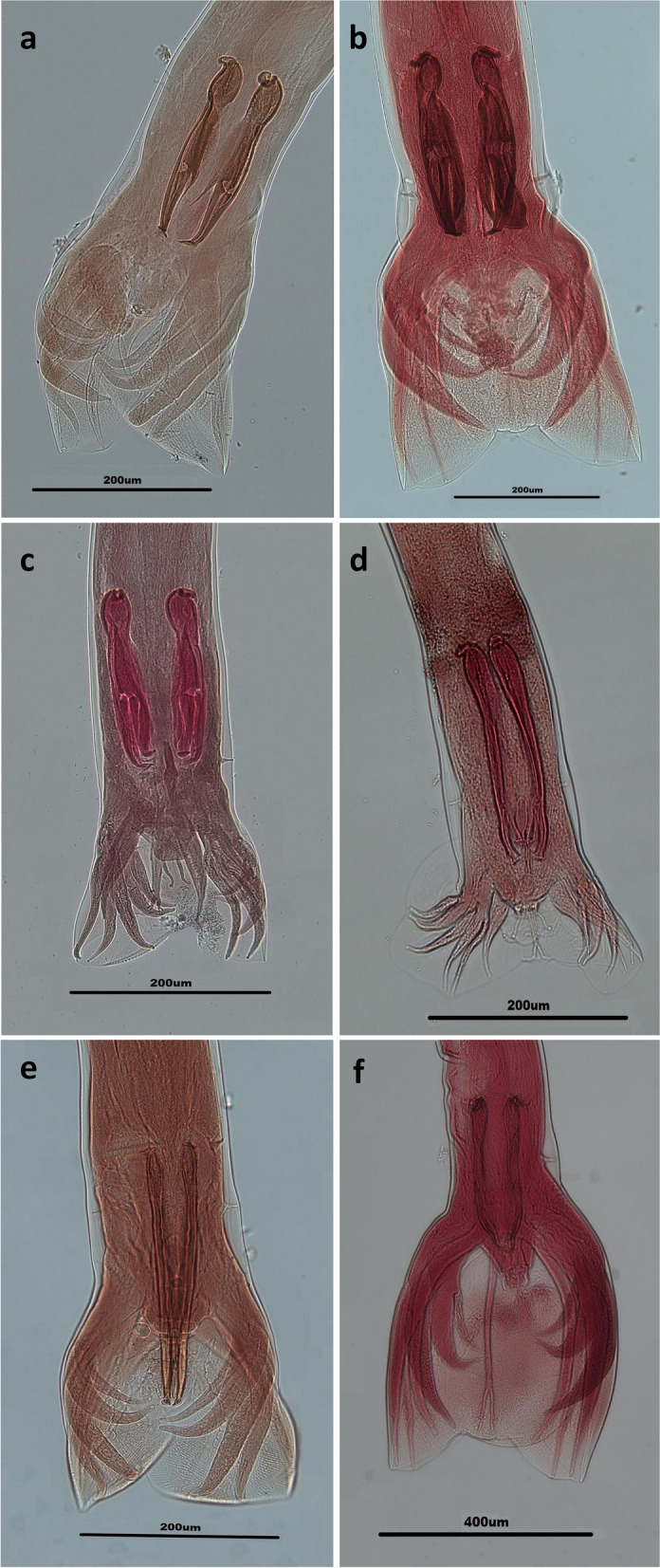
Light microscope view of *Marshallagia* and *Ostertagia* species isolated from ruminants in Guilan province, northern Iran. Posterior ends of males of *Ostertagia trifurcata* (**a**), *Marshallagia occidentalis* **(b**), *Ostertagia* *lyrata* (**c**), *Ostertagia ostertagi* (**d**), *Teladorsagia circumcincta* (**e**) and *Marshallagia marshalli* **(f**)

**Fig. 3:**
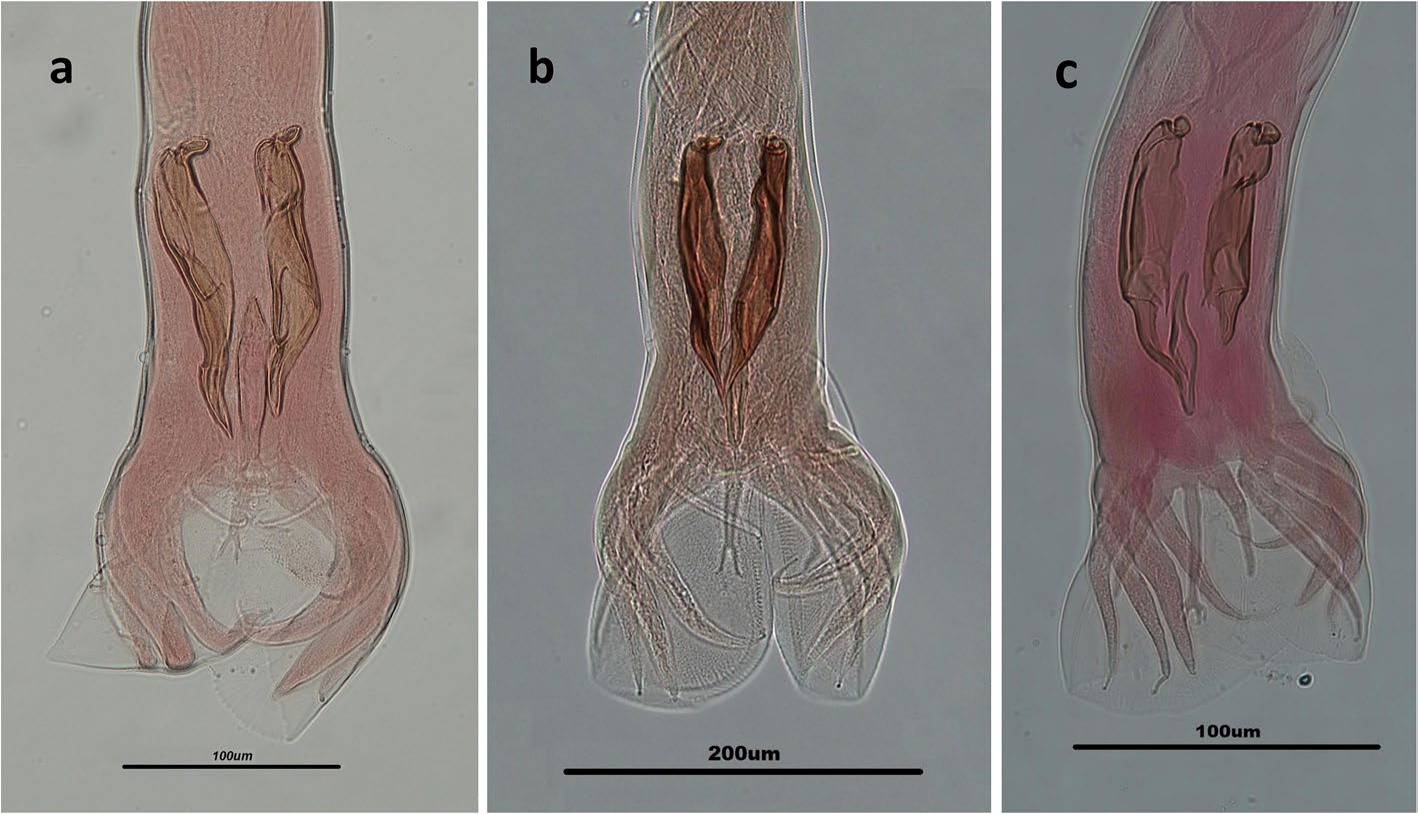
Light microscope view of *Trichostrongylus* species isolated from ruminants in Guilan province, northern Iran. Posterior ends of males of *Trichostrongylus colubriformis* (**a**), *Trichostrongylus vitrinus* (**b**) and *Trichostrongylus axei* (**c**)

**Fig. 4:**
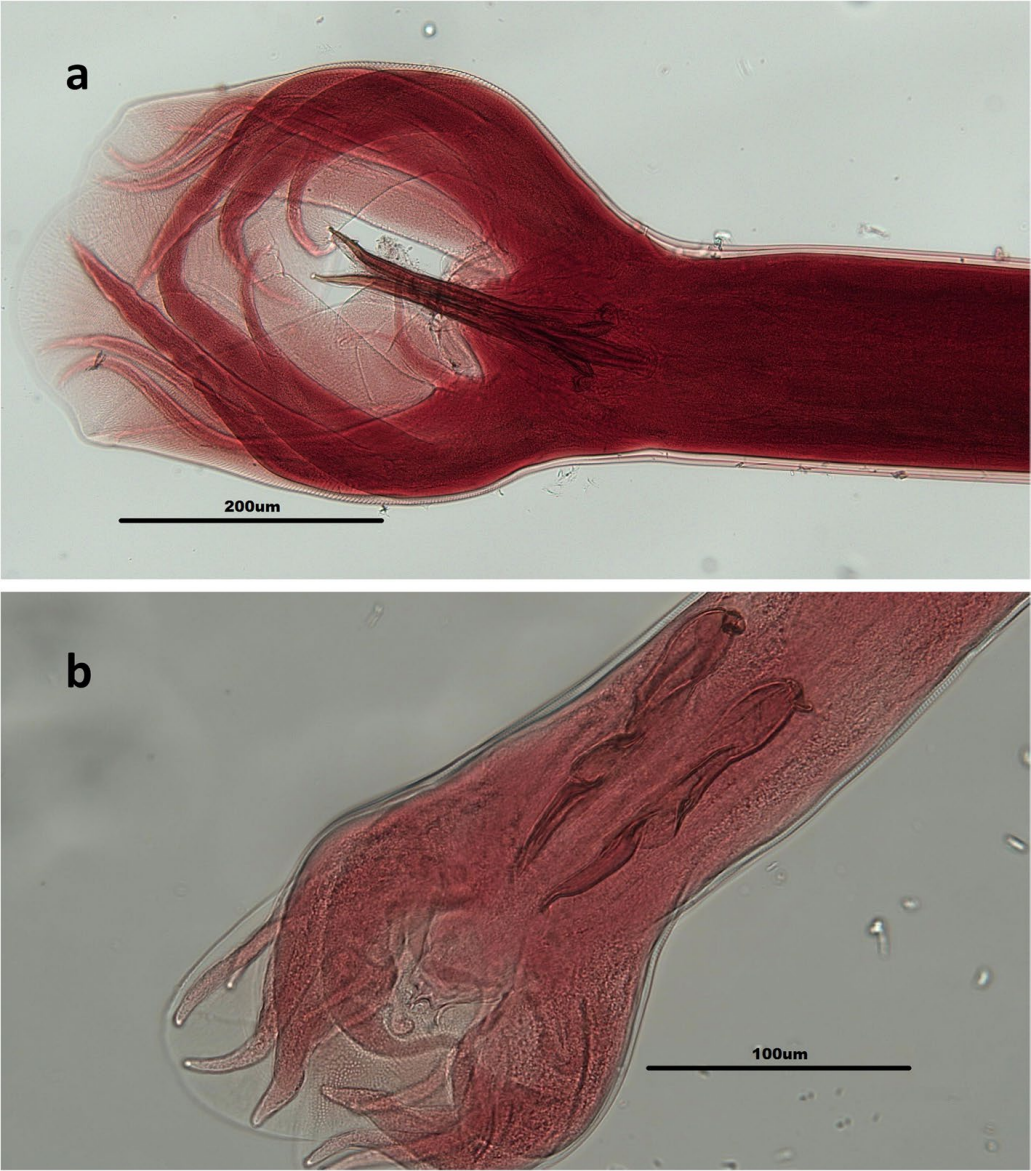
Posterior ends of males of *Haemonchus contortus* (**a**) and *Cooperia punctate* (**b**) isolated from ruminants in Guilan province, northern Iran

Overall, 83 out of the 144 ruminants (57.6%) were found infected with different genera of trichostrongyloid nematodes. The prevalence of total infections with the nematodes was 38.9% (28/72), 74.6% (44/59) and 84.6% (11/13) among cattle, sheep and goats, respectively. There are significant differences between the infection with trichostrongyloid nematodes and different kinds of ruminants (*P* < 0.0001). Eleven species of trichostrongylid nematodes including *H. contortus*, *M. marshalli*, *T. axei*, *T. colubriformis*, *T. vitrinus*, *O. trifurcata*, *T. circumcincta*, *M. occidentalis*, *O. lyrata*, *O. ostertagi*, and *C. punctate* were recovered from the ruminants (Figs. 2, 3, and 4).

The most prevalent trichostrongyloid nematodes in cattle and sheep were *O. ostertagi* (26.4%) and *M. marshalli* (64.4%), respectively. Also, the most predominant nematodes in goats were *T. circumcincta* (69.2%) and *M. marshalli* (53.8%). The prevalence rate of these parasites is shown in Table 1. Thirty-one of all ruminants (21.5%) were males and 113 (78.4%) were females. Of these, 18 (58.1%) of the males and 65 (57.5%) of the females were infected with the trichostrongyloid nematodes. No statistically significant difference was seen between total infection and the sex (*P* > 0.05). Also, there was not any significant difference between sex and the infection rates of each animal (*P* >0.05) (Table 2).

**Table 1:** Prevalence of Trichostrongyloid nematodes recovered from different livestock in Guilan province, northern Iran

Host (144)	Trichostrongyloid nematodes (%)											
	Hc	Mm	Mo	Tc	Ot	Ol	Oo	Cp	Ta	Tc	Tv	Co-infection
**Sheep (59)**	2 (3.4)	38 (64.4)	15 (25.4)	19 (32.2)	1 (1.7)	1 (1.7)	0 (0)	0 (0)	1 (1.7)	1 (1.7)	3 (5.1)	14 (19.4)
**Goat (13)**	2 (15.4)	7 (53.8)	0 (0)	9 (69.2)	1 (7.7)	1 (7.7)	1 (7.7)	0 (0)	3 (23.1)	2 (15.4)	2 (15.4)	28 (47.5)
**Cattle (72)**	0 (0)	2 (2.8)	0 (0)	0 (0)	0 (0)	6 (8.3)	19 (26.4)	9 (12.5)	6 (8.3)	0 (0)	0 (0)	8 (61.5)

*Abbreviations*: *Hc Haemonchus contortus*, *Mm Marshallagia marshalli*, *Mo Marshallagia occidentalis, Tc Teladorsagia circumcincta, Ot Ostertagia trifurcata*, *Ol Ostertagia lyrata*, *Oo Ostertagia ostertagi*, *Cp Cooperia punctate, Ta Trichostrongylus axei*, *Tc Trichostrongylus colubriformis*, *Tv Trichostrongylus vitrinus*

**Table 2:** Prevalence of infection with trichostrongyloid nematodes from different livestock in Guilan province, northern Iran according to sex

Hosts	Males		Femals		Total		***P***-value
	Positive N (%)	Negative N (%)	Positive N (%)	Negative N (%)	Positive N (%)	Negative N (%)	
**Sheep (59)**	8 (80)	2 (20)	36 (73.5)	13 (26.5)	44 (74.6)	15 (25.4)	1
**Goat (13)**	4 (100)	0 (0)	7 (77.8)	2 (22.2)	11 (84.6)	2 (15.4)	1
**Cattle (72)**	6 (35.3)	11 (64.7)	22 (40)	33 (60)	28 (38.9)	44 (61.1)	0.783
**Total (144)**	18 (58.1)	13 (41.9)	65 (57.5)	48 (42.5)	83 (57.6)	61 (42.4)	1

The genetic divergence within the specimens of *H. contortus*, *M. marshalli*, *T. axei*, *T. colubriformis*, *T. vitrinus* and *O. ostertagi* obtained from this study was 0%. However, intra-species distance for *O. trifurcate* and *O. circumcincta* isolates obtained in current study was 2.7 and 0.6%, respectively. The mean intra-species distance rate within species of *T. axei*, *T. colubriformis*, *T. vitrinus*, *O. ostertagi*, *M. marshalli*, *T. circumcincta*, *M. occidentalis*, *O. lyrata*, *H. contortus* and *C. punctate* obtained in this study and those available in the GenBank amounted to 0.2, 0.1, 0.4, 0.4, 0.3, 1.8, 0.2, 1.5, 0.3 and 1.2%, respectively.

**Fig. 5:**
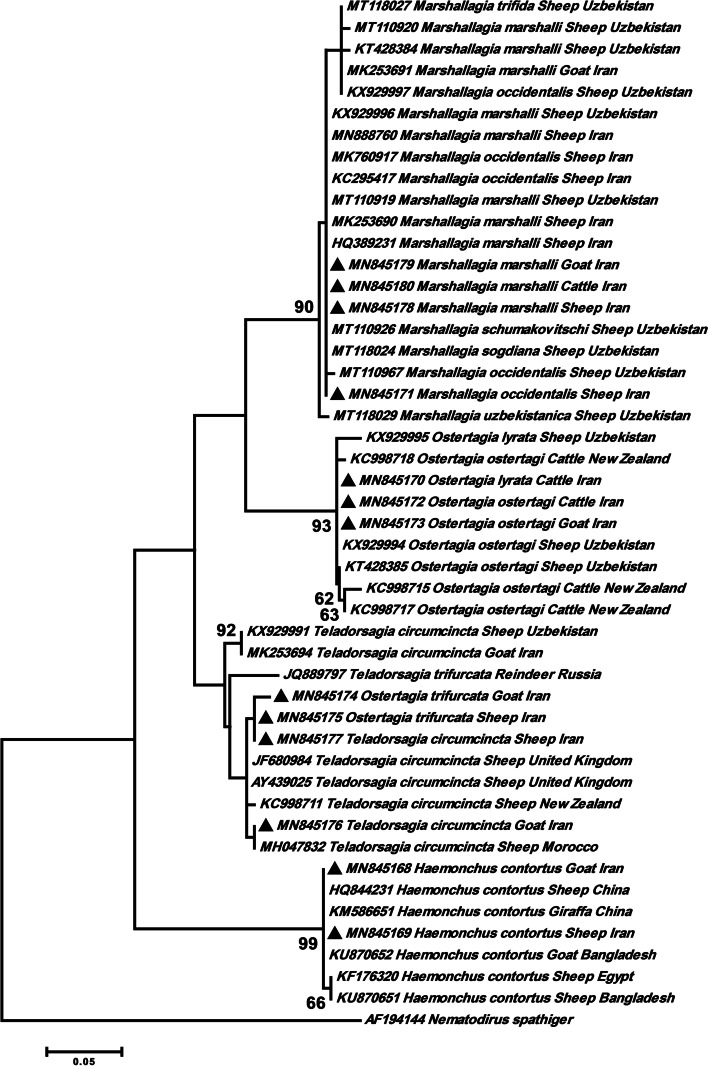
Phylogenetic tree of isolates of *Ostertagia*, *Marshallagia* and *Haemonchus* genera obtained in this study (▲) and other members of Haemonchidae family as retrieved from GenBank based on the partial ITS2 gene. The tree was constructed based on the maximum likelihood test and the Tamura 3-parameter model in MEGA6. *Nematodirus spathiger* sequence was used as the out group. Bootstrap values lower than 50 were omitted

The phylogenetic analysis of the Haemonchidae family illustrated that our *M. marshalli* (MN845179, MN845180 and MN845178) and *M. occidentalis* (MN845171) sequences placed together in one branch along with sheep isolates of *M. marshalli* from Iran (MN888760, MK253690 and HQ389231) and Uzbekistan (KX929996 and MT110919), and also sheep isolates of *M. occidentalis* from Iran (MK760917 and KC295417) and Uzbekistan (MT110967), and *M. schumakovitschi* (MT110926) and *M. sogdiana* (MT118024) from Uzbekistan. In addition, one goat isolate of *M. marshalli* from Iran (MK253691), sheep isolates of *M. marshalli* from Uzbekistan (MT110920 and KT428384) and sheep isolates of *M. trifida* (MT118027) and *M. occidentalis* (KX929997) from Uzbekistan clustered separately in this clade. Our sequences of *O. lyrata* (MN845170) and *O. ostertagi* (MN845172 and MN845173) grouped together in one clade along with sheep isolates of *O. ostertagi* (KX929994 and KT428385) and *O. lyrata* (KX929995) from Uzbekistan and cattle isolate of *O. ostertagi* (KX929995) from New Zealand. Two cattle of *O. ostertagi *isolates from New Zealand (KC998715 and KC998717) formed an innermost clade. The tree showed that our *T. circumcincta* isolates obtained from sheep (MN845177) and goat (MN845176), and *O. trifurcata* isolate from sheep (MN845175) clustered with sheep isolates of *T. circumcincta* from the United Kingdom (UK) (JF680984) and Morocco (MH047832)*.* Moreover, our goat isolate of *O. trifurcate* (MN845174) grouped with sheep isolates of *T. circumcincta* from New Zealand (KC998711) and the UK (AY439025). The *H. contortus* isolates (MN845168 and MN845169) clustered with sheep (HQ844231) and giraffe (KM586651) isolates from China and goat isolate from Bangladesh (KU870652). Two sheep sequences of *H. contortus* from Egypt (KF176320) and Bangladesh (KU870651) located separately in this clade (Fig. 5).

**Fig. 6:**
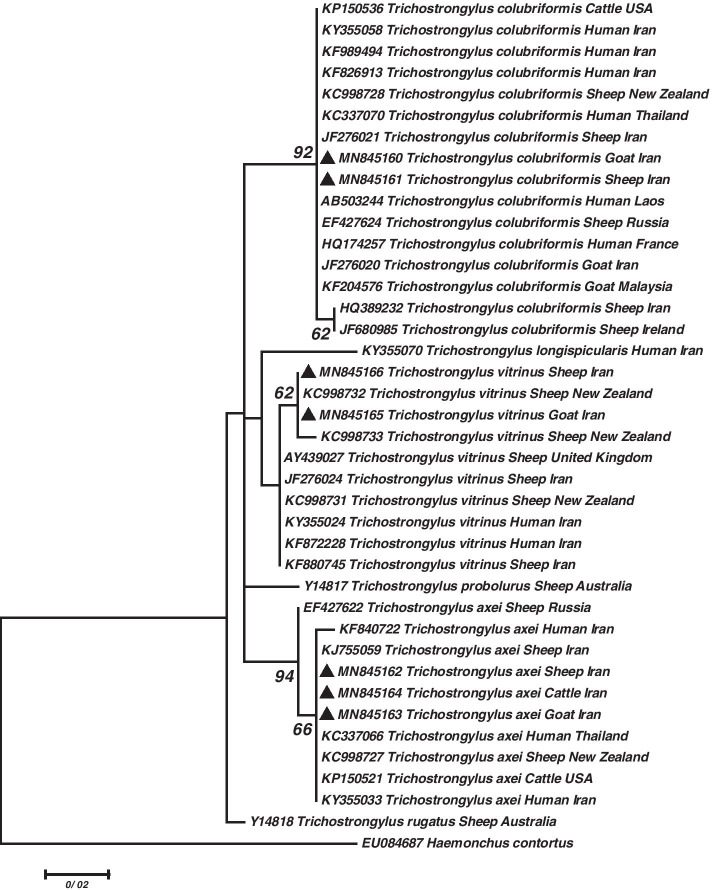
Phylogenetic tree of isolates of the *Trichostrongylus* species obtained in this study (▲) and other members of Trichostrongylidae family as retrieved from GenBank based on the partial ITS2 gene. The tree was constructed based on the maximum likelihood test and the Tamura 3-parameter model in MEGA6. *Haemonchus contortus* sequence was used as the out group. Bootstrap values lower than 50 were omitted

The phylogenetic reconstruction of Trichostrongylidae family showed that our sequences of *T. colubriformis* obtained from sheep (MN845161) and goat (MN845160) clustered with *T. colubriformis* isolated from human (KY355058, KF989494 and KF826913), sheep (JF276021), and goat (JF276020) in Iran. They also grouped with human isolates of *T. colubriformis* from France (HQ174257), Laos (AB503244), and Thailand (KC337070), sheep isolates from New Zealand (KC998728) and Russia (EF427624), goat isolate from Malaysia (KF204576), and cattle isolate from the United States of America (USA) (KP150536). In this clade, two sheep sequences of *T. colubriformis* from Iran (HQ389232) and Ireland (JF680985) located separately. The tree illustrated our sheep (MN845166) and goat (MN845165) isolates of *T. vitrinus* grouped with sheep isolates of *T. vitrinus* from New Zealand (KC998732 and KC998733). Moreover, our *T. axei* sequences isolated from sheep (MN845162), goat (MN845163), and cattle (MN845164) clustered with human isolates from Iran (KY355033 and KF840722) and Thailand (KC337066), sheep isolates from New Zealand (KC998727) and Iran (KJ755059), and cattle from the USA (KP150521) (Fig. 6).

**Fig. 7:**
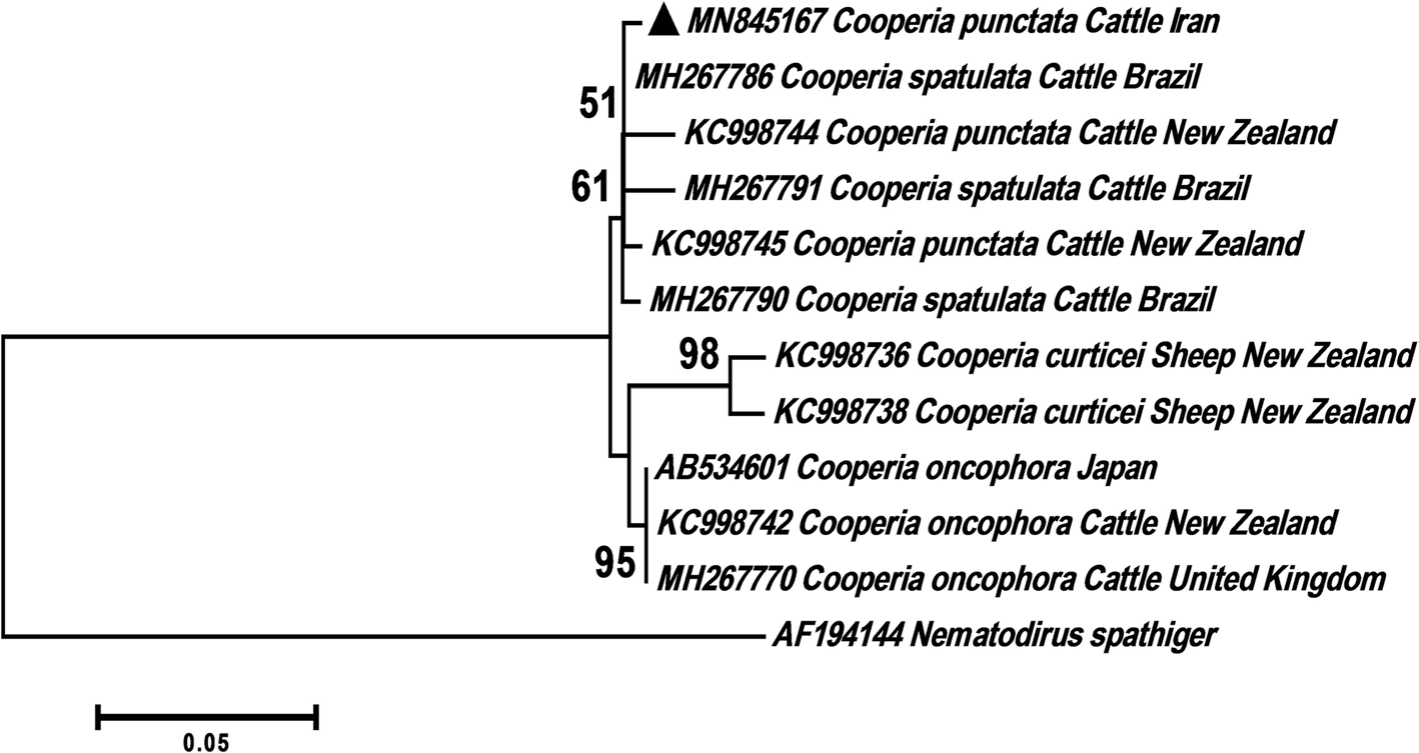
Phylogenetic tree of isolates of the *Cooperia punctata* obtained in this study (▲) and other members of Cooperiidae family as retrieved from GenBank based on the partial ITS2 gene. The tree was constructed based on the maximum likelihood test and the Tamura 3-parameter model in MEGA6. *Nematodirus spathiger* sequence was used as the out group. Bootstrap values lower than 50 were omitted

The phylogenetic analysis of the Cooperiidae family showed that our *C. punctata* (MN845167) obtained from cattle grouped with cattle isolates of *C. punctata* from New Zealand (KC998744 and KC998745) and cattle isolates of *C. spatulata* from Brazil (MH267786, MH267791 and MH267790) (Fig. 7).

## Discussion

This study revealed 38.9% cattle, 74.6% sheep, and 84.6% goats were infected with the trichostrongyloid nematodes. The prevalence of abomasal nematodes were reported 44 and 25% of the slaughtered cows in Tabriz, northwestern Iran [26] and Kerman southeastern Iran [27], respectively. Moreover, the prevalence of trichostrongyloid nematodes was reported 94 and 6.1% of the cattle in Belgium [28] and Ethiopia [29], respectively. Several studies from Iran showed high infection rate of the parasites in sheep and goats. Kordi et al. [27] reported a high infection rate as 100% in sheep and goats in Kerman. Also, Garedaghi et al. [30] found the infection rates of 81 and 71.2% in sheep and goats of Mazanderan province, respectively. However, on the contrary to the present study, some previous studies in Iran have reported lower infection rate in the small ruminants [31, 32]. Furthermore, Fufa et al. [33] reported the prevalence of abomasum nematodes in sheep and goats, slaughtered in Ethiopia to be 83.6 and 77.6%, respectively.

Our finding showed that the most predominant trichostrongyloid nematode in cattle in the study area was *O. ostertagi* (26.4%). *O. ostertagi* is a common parasitic nematode of cattle in the world, but it can also be found to a lesser extent in sheep, goats, wild ruminants, and horses [34]. Similar to our study, *O. ostertagi* was reported as predominant nematode in cattle in Canada [35]. However, *H. contortus* was reported in cattle with highest prevalence (17.6%) in Tabriz, northwestern of Iran [26]. Abuhay et al. [29] reported *T. axei* (2.86%) was the most predominant abomasal nematodes of cattle slaughtered in Ethiopia.

In present study, *M. marshalli* was identified as the most predominant nematode in sheep (64.4%). Also, the most prevalent nematodes in goats were *T. circumcincta* (69.2%) and *M. marshalli* (53.8%). Similar to our results, Pestechian et al. [36] reported that *M. marshalli* (57.1%) in sheep and *T. circumcincta* (47.2%) in goat were dominant species in Isfehan, central part of Iran. *M. marshalli* was also reported as more prevalent species among both sheep (20.5%) and goats (60%) in Qazvin province, northwestern of the country [37]. In other studies, in Iran, *H. contortus* in goats and *T. circumcincta* in sheep were also found as the frequently reported species in northwest and west of the country, respectively [31, 38]. Aligned with our findings, *M. marshalli* occurred with the highest prevalence among sheep in Canada [39]. In other study in Ethiopia, *Haemonchus* spp. were found as most predominant abomasal nematodes in both sheep and goats [40]. Recently, Ashrafi et al. [7] reported the occurrence of natural human infection by *T. circumcincta* for the first time in Iran and the second time in the world.

In the current study, *Trichostrongylus* species including *T. colubriformis*, *T. axei*, and *T. vitrinus* were detected from the animals. Previously, several studies reported these nematodes as the predominant species of *Trichostrongylus* among different herbivores in most parts of Iran [9]. Recently, infection with some species of *Trichostrongylus* including *T. colubriformis*, *T. axei*, *T. vitrinus*, and *T. longispicularis* have been reported from humans in Guilan province from which *T. colubriformis* was as the most common species [12]. Additionally, Gholami et al. [41] and Sharifdini et al. [17] reported *T. colubriformis* as the most probable common species and *T. axei* in inhabitants of Mazandaran province, north of Iran. Also, *T. colubriformis* was identified as a predominant species among humans in Thailand, France, and Laos [11, 16, 42].

In this study, *O. lyrata* was reported from cattle (8.3%), sheep (1.7%) and goats (7.7%). Until now, *O. lyrata* has not been reported from Iran, and to best of our knowledge this study is the first report of this species in the ruminants. This nematode has mainly reported from cattle in different parts of the world [43–45].

In the current study, there was no significant difference between trichostrongyloid infection and the sex (*P* > 0.05). Our findings are in consistent with Kordi et al. [27], Garedaghi et al. [31] and Amniattalab et al. [46]. Nevertheless, some researchers have observed higher rates of trichostrongyloid nematode infections in female hosts compared to the males [47, 48].

In the last few decades, PCR-sequencing technique was applied to assess the taxonomic identification, diversity, and phylogenetic relationships among trichostrongyloid species. Some investigations showed that the ITS2 region of rRNA gene is useful tool for identification and phylogenetic analysis of trichostrongyloid nematodes [7, 12, 17, 41]. In the present study, no intra-generic differences were apparent between *H. contortus*, *M. marshalli*, *T. axei*, *T. colubriformis*, *T. vitrinus,* and *O. ostertagi* isolated from different domestic animals, but intra- species variations were noted within *O. trifurcate* and *O. circumcincta* isolates being 2.7 and 0.6%, respectively. The inter-species differences between species of genera *Ostertagia*, *Marshallagia*, *Trichostrongylus* obtained in our study were 1.00–13.1%, 0.1–1.5% and 2–6.4%.

Our phylogenetic analysis of Haemonchidae family showed that different species of *Marshallagia* including *M. marshalli, M. occidentalis, M. schumakovitschi*, *M. sogdiana* and *M. trifida* were grouped together in one cluster. Additionally, *M. uzbekistanica* placed separately from other species of *Marshallagia* with strong support*.* Our finding showed that the ITS2 gene is not suitable for diagnosis and gene diversity of *Marshallagia* species so that *M. marshalli, M. occidentalis* obtained in this study had 100% identity. On the other hand, the phylogenetic analysis showed that *O. lyrata* with *O. ostertagi* and *T. circumcincta* with *O. trifurcata* grouped together in one clade. The presented data indicated that the ITS2 sequence is uninformative for phylogenetic inference among the species, because of the lack of sufficient diversity within the gene. In this line, Zarlenga et al. [21] illustrated that the ITS1 is inappropriate in resolving phylogenetic issues within genus *Ostertagia*. Thus, the application of additional genetic data is necessary for exploring the phylogenetic relationships among the species. *H. contortus* as other genus of Haemonchidae family, was well separated in the tree, with strong support. Our study also revealed *H. contortus* sequences obtained from goat and sheep had 100% similarity. Several studies showed that the ITS2 is a useful target for identification of two species within the genus *Haemonchus* and the intraspecific variation within *H. contortus* [49–52].

The existence of genetic diversity of the ITS2 fragment among different *Trichostrongylus* species has been confirmed previously [7, 12, 17, 41]. The phylogenetic tree of Trichostrongylidae family represented that all species were clearly separated and they were similar to the species obtained from human and animal subjects in Iran and other countries. Our study also showed that *T. colubriformis*, *T. vitrinus,* and *T. axei* isolates obtained from different animals had 100% similarity. Sequences of *Trichostrongylus* species isolated in present study had 99–100% homology with those obtained from humans and domestic animals in Iran. This finding confirmed that the ruminant and human *Trichostrongylus* species have close phylogenetic relationship. This could explain that human infections in the study area may occur due to close contact with domestic animals and application of domestic animal’s dung as fertilizer in vegetable farms.

Our phylogenetic analysis of the Cooperiidae family showed that isolates of *C. punctata* and *C. spatulata* clustered together in one clade. This finding presented that the ITS2 fragment was unreliable for differentiation at the species level. Recently, Ramünke et al. [53] reported that the ITS2 has minimal nucleotide differences between the *Cooperia* species and is accurate only up to genus level identification. The authors also showed that, in contrast to the morphological characterization, no molecular distinction between *C. punctata* and *C. spatulata* was possible with any of the four gene markers including isotype 1 β-tubulin, the mitochondrial *Cox*2, ITS, and mitochondrial 12S rRNA genes [53].

## Conclusions

The findings of this study showed the high prevalence and species diversity of trichostrongyloid nematodes in different ruminants of northern Iran. Therefore, implement antiparasitic strategies seems to be necessary to increase productivity of the domestic animals. Furthermore, extra epidemiological studies in different seasons and reigns of the country should be conducted to provide more information about the seasonal dynamics of the gastrointestinal helminths. The present molecular analysis of the ITS2 fragment is not sufficient for valid species identification of Haemonchidae and Cooperiidae families and only can differentiate members of Trichostrongylidae family. Providing more genetic markers alongside obtaining specimens from other geographical locations and hosts would be valuable for better understanding of their phylogenetic relationships.

## Data Availability

All data generated or analyzed during the present study are included in this published article.

## Abbreviations

ITS*-*rDNA: Internal transcribed spacer ribosomal DNA
*Cox*1: Cytochrome c oxidase 1
PCR: Polymerase chain reaction

## References

[1] Karshima SN, Maikai BV, Kwaga JKP (2018). Helminths of veterinary and zoonotic importance in Nigerian ruminants: a 46-year meta-analysis (1970-2016) of their prevalence and distribution. Infect Dis Poverty.

[2] Perry BD (2002). Investing in animal health research to alleviate poverty: ILRI (aka ILCA and ILRAD).

[3] Krecek RC, Waller PJ (2006). Towards the implementation of the “basket of options” approach to helminth parasite control of livestock: emphasis on the tropics/subtropics. Vet Parasitol.

[4] Besier RB, Kahn LP, Sargison ND, Van Wyk JA (2016). Diagnosis, treatment and Management of Haemonchus contortus in small ruminants. Adv Parasitol.

[5] Greenland K, Dixon R, Khan SA, Gunawardena K, Kihara JH, Smith JL (2015). The epidemiology of soil-transmitted helminths in Bihar state, India. PLoS Negl Trop Dis.

[6] Zajac AM, Garza J (2020). Biology, epidemiology, and control of gastrointestinal nematodes of small ruminants. Vet Clin North Am Food Anim Pract.

[7] Ashrafi K, Sharifdini M, Heidari Z, Rahmati B, Kia EB (2020). Zoonotic transmission of *Teladorsagia circumcincta* and *Trichostrongylus* species in Guilan province, northern Iran: molecular and morphological characterizations. BMC Infect Dis.

[8] Sato M, Yoonuan T, Sanguankiat S, Nuamtanong S, Pongvongsa T, Phimmayoi I (2011). Short report: human *Trichostrongylus colubriformis* infection in a rural village in Laos. Am J Trop Med Hyg..

[9] Ghadirian E, Arfaa F (1975). Present status of trichostrongyliasis in Iran. Am J Trop Med Hyg.

[10] Muller R (2002). Worms and human disease.

[11] Watthanakulpanich D, Pongvongsa T, Sanguankiat S, Nuamtanong S, Maipanich W, Yoonuan T (2013). Prevalence and clinical aspects of human *Trichostrongylus colubriformis* infection in Lao PDR. Acta Trop.

[12] Sharifdini M, Derakhshani S, Alizadeh SA, Ghanbarzadeh L, Mirjalali H, Mobedi I (2017). Molecular identification and phylogenetic analysis of human *Trichostrongylus* species from an endemic area of Iran. Acta Trop.

[13] Ghanbarzadeh L, Saraei M, Kia EB, Amini F, Sharifdini M (2019). Clinical and haematological characteristics of human trichostrongyliasis. J Helminthol.

[14] Ashrafi K, Tahbaz A, Sharifdini M, Mas-Coma S (2015). Familial *Trichostrongylus* infection misdiagnosed as acute fascioliasis. Emerg Infect Dis.

[15] Ghadirian E, Arfaa F (1973). First report of human infection with *Haemonchus contortus*, *Ostertagia ostertagi*, and *Marshallagia marshalli* (family Trichostrongylidae) in Iran. J Parasitol.

[16] Phosuk I, Intapan PM, Sanpool O, Janwan P, Thanchomnang T, Sawanyawisuth K (2013). Molecular evidence of *Trichostrongylus colubriformis* and *Trichostrongylus axei* infections in humans from Thailand and Lao PDR. Am J Trop Med Hyg..

[17] Sharifdini M, Heidari Z, Hesari Z, Vatandoost S, Kia EB (2017). Molecular Phylogenetics of *Trichostrongylus* species (Nematoda: Trichostrongylidae) from humans of Mazandaran Province, Iran. Korean J Parasitol.

[18] Kandil OM, Abdelrahman KA, Fahmy HA, Mahmoud MS, El Namaky AH, Miller JE (2017). Phylogenetic patterns of *Haemonchus contortus* and related trichostrongylid nematodes isolated from Egyptian sheep. J Helminthol.

[19] Ghasemikhah R, Sharbatkhori M, Mobedi I, Kia E, Harandi MF, Mirhendi H (2012). Sequence analysis of the second internal transcribed spacer (ITS2) region of rDNA for species identification of *Trichostrongylus* nematodes isolated from domestic livestock in Iran. Iran J Parasitol.

[20] Sun MM, Han L, Zhang FK, Zhou DH, Wang SQ, Ma J (2018). Characterization of the complete mitochondrial genome of *Marshallagia marshalli* and phylogenetic implications for the superfamily Trichostrongyloidea. Parasitol Res.

[21] Zarlenga DS, Hoberg EP, Stringfellow F, Lichtenfels JR (1998). Comparisons of two polymorphic species of *Ostertagia* and phylogenetic relationships within the Ostertagiinae (Nematoda: Trichostrongyloidea) inferred from ribosomal DNA repeat and mitochondrial DNA sequences. J Parasitol..

[22] Kazemi Rad L, Mohammadi H (2015). Climate change assessment in gilan province, Iran. Int J Agric Sci.

[23] Anderson RC (2000). Nematode parasites of vertebrates: their development and transmission.

[24] Skrjabin K, Shikhobalova N, Shul'ts R (1954). Essentials of nematodology. Trichostrongyloids of animals and man Vol. III Pub.

[25] Chilton NB (2004). The use of nuclear ribosomal DNA markers for the identification of bursate nematodes (order Strongylida) and for the diagnosis of infections. Anim Health Res Rev.

[26] Mashayekhi M, Gharedaghi Y, Farazmand MR (2013). Study of abomasal nematodes in adult cattles in abattoir of Tabriz Iran. Bull Env Pharmacol Life Sci.

[27] Kordi B, Mirzaei M, Nooshadokht M (2019). Identification of abomasum nematodes fauna of ruminants in Kerman industrial slaughterhouse, Iran. Biomed J Sci Tech Res.

[28] Agneessens J, Claerebout E, Dorny P, Borgsteede FH, Vercruysse J (2000). Nematode parasitism in adult dairy cows in Belgium. Vet Parasitol.

[29] Abuhay M, Hamid M, Tintagu T (2017). Species composition and status of abomasal nematodes of cattle slaughtered at Abergelle export abattoir, Mekelle, Ethiopia. Glob Vet.

[30] Garedaghi Y, Hashemzadefarhang H, Esmaeli A (2013). Study on the prevalence and species composition of abomasal nematodes in small ruminants slaughtered at Behshahr town. Iran J Vet Adv.

[31] Garedaghi Y, Hashemzadefarhang H, Fattahi A (2013). Prevalence of abomasal nematodes in sheep slaughtered at Baneh town. Am J Anim Vet Sci.

[32] Nabavi R, Eslami A, Shokrani H, Bokaie S, Shayan P, Saadati D (2011). Study on the prevalence, intensity and seasonal dynamics of abomasal helminths in sheep from different climatic zones of Iran. World Appl Sci J.

[33] Fufa A, Ephrem T, Bersisa K, Bekele M, Alemayehu R, Etana D (2009). Abomasal nematodes: prevalence in small ruminants slaughtered at Bishooftu town, Ethiopia. Vet Med Int.

[34] Fox M (1993). Pathophysiology of infection with *Ostertagia ostertagi* in cattle. Vet Parasitol.

[35] Slocombe J (1974). Abomasal nematodes in cattle in Ontario. Can J Comp Med.

[36] Pestechian N, Kalani H, Faridnia R, Yousefi H-A (2014). Zoonotic gastrointestinal nematodes (Trichostrongylidae) from sheep and goat in Isfahan, Iran. Acta Sci Vet.

[37] Barghandan T, Hajialilo E, Sharifdini M, Javadi A (2019). Prevalence and phylogenetic analysis of gastrointestinal helminths (Nematoda: Trichostrongylidae) in ruminant livestock of Northwest Iran. Vet Fak Derg.

[38] Hakimzadegan M, Khosroshahi MK (2013). Prevalence of abomasal nematodes in slaughtered goats at industrial Urmia slaughterhouse, West Azerbaijan Province, Northwest of Iran. J Poult Sci.

[39] Aleuy OA, Ruckstuhl K, Hoberg EP, Veitch A, Simmons N, Kutz SJ (2018). Diversity of gastrointestinal helminths in Dall's sheep and the negative association of the abomasal nematode, *Marshallagia marshalli*, with fitness indicators. PLoS One.

[40] Thomas N, Teshale S, Kumsa B (2007). Abomasal nematodes of sheep and goats slaughtered in Awassa (Ethiopia): species composition, prevalence and vulvar morphology. Helminthologia..

[41] Gholami S, Babamahmoodi F, Abedian R, Sharif M, Shahbazi A, Pagheh A (2015). *Trichostrongylus colubriformis*: possible most common cause of human infection in Mazandaran province, north of Iran. Iran J Parasitol.

[42] Lattes S, Ferte H, Delaunay P, Depaquit J, Vassallo M, Vittier M (2011). *Trichostrongylus colubriformis* nematode infections in humans, France. Emerg Infect Dis.

[43] Lichtenfels J, Pilitt P, Lancaster M (1988). Systematics of the nematodes that cause ostertagiasis in cattle, sheep and goats in North America. Vet Parasitol.

[44] Karbowiak G, Demiaszkiewicz A, Pyziel A, Wita I, Moskwa B, Werszko J (2014). The parasitic fauna of the European bison (*bison bonasus*) (Linnaeus, 1758) and their impact on the conservation. Part 1 the summarising list of parasites noted. Acta Parasitol.

[45] McKenna P (1997). Checklist of helminth parasites of terrestrial mammals in New Zealand. N Z J Zool.

[46] Amniattalab A, Rasouli S, Saebnajjar M (2014). Seasonal prevalence and pathological changes of Ostertagiasis in abomasum of slaughtered sheep in Khoy city in Iran. Adv Environ Biol.

[47] Lateef M, Iqbal Z, Jabbar A, Khan MN, Akhtar MS (2005). Epidemiology of trichostrongylid nematode infections in sheep under traditional husbandry system in Pakistan. Int J Agric Biol.

[48] Demissie T, Tesfaye D, Fekadu A, Asefa I (2013). Study on abomasal nematodes of sheep and goats: comparison and characterization of vulvar morphology of *Haemonchus* in Hawassa, Ethiopia. Afr J Agric Res.

[49] Laosutthipong C, Eardmusic S (2019). Genetic characterization of *Haemonchus contortus* from slaughtered goats in Cha-Am District, Phetchaburi Province, Thailand. Songklanakarin J Sci Technol.

[50] Shen D-d, Wang J-f, D-y Z, Z-w P, Yang T-y, Wang Z-d, et al. Genetic diversity of *Haemonchus contortus* isolated from sympatric wild blue sheep (*Pseudois nayaur*) and sheep in Helan Mountains, China. Parasit Vectors. 2017;10(1):437. 10.1186/s13071-017-2377-0 PMC5606089 28927469

[51] Troell K, Mattsson JG, Alderborn A, Höglund J (2003). Pyrosequencing™ analysis identifies discrete populations of *Haemonchus contortus* from small ruminants. Int J Parasitol Parasites.

[52] Gasser RB, Zhu X, Chilton NB, Newton LA, Nedergaard T, Guldberg P (1998). Analysis of sequence homogenisation in rDNA arrays of *Haemonchus contortus* by denaturing gradient gel electrophoresis. Electrophoresis..

[53] Ramünke S, de Almeida BF, von Son-de FE, von Samson-Himmelstjerna G, Krücken J (2018). Molecular marker sequences of cattle *Cooperia* species identify *Cooperia spatulata* as a morphotype of *Cooperia punctata*. PLoS One.

